# Ferritin Cutoffs and Diagnosis of Iron Deficiency in Primary Care

**DOI:** 10.1001/jamanetworkopen.2024.25692

**Published:** 2024-08-05

**Authors:** Levy Jäger, Yael Rachamin, Oliver Senn, Jakob M. Burgstaller, Thomas Rosemann, Stefan Markun

**Affiliations:** 1 Institute of Primary Care, University Hospital Zurich, University of Zurich, Zurich, Switzerland; 2 Campus Stiftung Lindenhof Bern (SLB), Bern, Switzerland

## Abstract

**Importance:**

Ferritin is often measured by general practitioners, but the association of different cutoffs with the rates of iron deficiency diagnoses, particularly nonanemic iron deficiency, is unknown.

**Objective:**

To investigate the association of the ferritin cutoff choice with the incidence of nonanemic and anemic iron deficiency diagnoses in primary care.

**Design, Setting, and Participants:**

In this retrospective cohort study, patients 18 years or older with at least 1 consultation with a general practitioner participating in the Family Medicine Research Using Electronic Medical Records (FIRE) project, an electronic medical records database of Swiss primary care, from January 1, 2021, to November 30, 2023, were evaluated.

**Exposures:**

Sex, age, clinical patient characteristics, and professional general practitioner characteristics.

**Main Outcomes and Measures:**

Incidence of iron deficiency diagnoses (nonanemic and anemic) at ferritin cutoffs of 15, 30, and 45 ng/mL and ferritin testing itself. Time-dependent Cox proportional hazards regression was used to examine associations of patient and general practitioner characteristics with ferritin testing as adjusted hazard ratios (AHRs).

**Results:**

The study included 255 351 patients (median [IQR] age, 52 [36-66] years; 52.1% female). Per 1000 patient-years and at ferritin cutoffs of 15, 30, and 45 ng/mL, iron deficiency diagnoses had incidences of 10.9 (95% CI, 10.6-11.2), 29.9 (95% CI, 29.4-30.4), and 48.3 (95% CI, 47.7-48.9) cases, respectively; nonanemic iron deficiency diagnoses had incidences of 4.1 (95% CI, 3.9-4.2), 14.6 (95% CI, 14.3-15.0), and 25.8 (95% CI, 25.3-26.2) cases, respectively; and anemic iron deficiency diagnoses had incidences of 3.5 (95% CI, 3.3-3.7), 6.0 (95% CI, 5.8-6.2), and 7.5 (95% CI, 7.3-7.7) cases, respectively. Ferritin testing showed notable associations with fatigue (AHR, 2.03; 95% CI, 1.95-2.12), anemia (AHR, 1.75; 95% CI, 1.70-1.79), and iron therapy (AHR, 1.50; 95% CI, 1.46-1.54). Ferritin testing was associated with female sex in all age groups, including postmenopausal. Of the patients who received ferritin testing, 72.1% received concomitant hemoglobin testing, and 49.6% received concomitant C-reactive protein testing.

**Conclusions and Relevance:**

In this retrospective cohort study of primary care patients, ferritin cutoffs of 30 and 45 ng/mL were associated with a substantially higher incidence of iron deficiency compared with 15 ng/mL. These results provide a basis for health system-level evaluation and benchmarking of ferritin testing in high-resource settings and call for a harmonization of diagnostic criteria for iron deficiency in primary care.

## Key Points

**Question:**

How is the choice of guideline-recommended cutoffs for ferritin associated with the incidence of iron deficiency diagnoses in primary care?

**Findings:**

In this cohort study of 255 351 adult primary care patients, ferritin cutoffs of 15, 30, and 45 ng/mL were associated with incidences of iron deficiency diagnoses of 10.9, 29.9, and 48.3 cases per 1000 patient-years, respectively.

**Meaning:**

The results of this study provide useful components for the evaluation of ferritin testing in high-resource primary care settings and call for harmonization of guidelines for iron deficiency.

## Teaser

This cohort study of adult primary care patients estimates how the incidence of diagnoses of nonanemic and anemic iron deficiency is associated with the choice of ferritin cutoff.

## Introduction

Iron deficiency is a common condition and a leading cause of years lived with disability worldwide, mainly due to subsequent anemia.^1^ A depletion of iron stores that does not yet result in anemia, simply referred to as nonanemic iron deficiency, has recently gained attention as a distinct clinical entity.^2^ Associated with various symptoms, including fatigue, restless legs syndrome, and hair loss,^3^ nonanemic iron deficiency has been estimated to be more common than anemic iron deficiency.^2^

Measurement of (serum) ferritin is considered the mainstay of iron deficiency diagnosis,^4^ and ferritin is a commonly requested laboratory test in various high-resource primary care settings.^5,6,7^ In particular, a Swiss study revealed that 27% of the population received serum ferritin testing in 2018,^8^ and ferritin ranked among the most frequently ordered laboratory tests in Swiss primary care between 2009 and 2018.^9^ At the same time, iron deficiency guidelines are conflicting as to which populations benefit most from ferritin testing.^4^ For example, the US Preventive Services Task Force specifically mentions children and pregnant women^10^ but makes no recommendation for the general population. A prominent Swiss primary care guideline discourages iron deficiency screening in the general population, with exceptions for conditions that require special consideration due to increased risk, such as inflammatory bowel disease (IBD), or when evidence suggests a benefit from replete iron stores, such as chronic kidney disease (CKD).^11^

The optimal ferritin cutoffs for the diagnosis of iron deficiency, especially nonanemic iron deficiency, are controversial. Different guidelines suggest widely varying cutoffs ranging from 12 to 15 ng/mL through 25 to 30 ng/mL to 45 to 50 ng/mL in the general population (to convert to micrograms per liter, multiply by 1).^4^ The choice of the ferritin cutoff may have important implications. On the one hand, choosing too low a cutoff could result in withholding therapy from patients affected by the negative health consequences of iron deficiency. On the other hand, choosing too high a cutoff could lead to overtreatment of patients who do not benefit from iron therapy, with potential harm from adverse effects (especially with oral preparations^12^) and an unjustified waste of health care resources.

Although previous studies have estimated incidences of anemic iron deficiency in primary care,^13,14^ they have not evaluated the choice of different ferritin cutoffs, and the incidence of nonanemic iron deficiency has not been investigated. However, appropriate data would be essential for a comprehensive evaluation of ferritin testing in primary care. Furthermore, little is known about the factors associated with ferritin testing. Our study aims to fill these gaps by estimating how the incidence of diagnoses of nonanemic and anemic iron deficiency depends on the choice of ferritin cutoff and by examining the determinants of ferritin testing in Swiss primary care.

## Methods

### Setting and Data Source

We conducted a retrospective cohort study using data from the Family Medicine Research Using Electronic Medical Records (FIRE) project, a database of routine data from Swiss general practitioners hosted by the Institute of Primary Care of the University of Zurich.^15^ The database contains anonymized data on medication prescriptions, including Anatomical Therapeutic Chemical codes^16^ and Global Trade Item Numbers, codes of the International Classification of Primary Care (ICPC-2) system,^17^ body height and weight, sex and year of birth, practice postal code, and laboratory test results.

The local ethics committee of the Canton of Zurich waived ethical approval for this study and the need for informed consent because data from the FIRE project fall outside the scope of the *Federal Act on Research involving Human Beings*.^18^ We report the results of this study according to the Reporting of Studies Conducted Using Observational Routinely-Collected Health Data (RECORD) guidelines (eTable 1 in Supplement 1).^19^

### Study Population

We conducted a retrospective cohort study from January 1, 2021, to November 30, 2023, including all patients with at least 1 consultation during the study period. For each patient, we used the date of the first consultation during the study period as the time of inclusion and excluded all patients younger than 18 years at inclusion to obtain the study cohort.

### Explanatory Covariates

We defined sex-age strata (combinations of female and male and age <25, 25-34, 35-44, 45-54, 55-64, 65-74, and ≥75 years) and identified the presence of the following clinical factors often mentioned in guidelines as requiring special considerations for ferritin testing^4^: CKD, IBD, rheumatic diseases, congestive heart failure, pregnancy, cancer, proton pump inhibitor (PPI) therapy, and fatigue (see eTable 2 in Supplement 1 for the operationalized criteria). We further identified anemia as the presence of hemoglobin concentrations less than 12 g/dL for women and less than 13 g/dL for men (to convert to grams per liter, multiply by 10) according to the World Health Organization definition,^20^ iron therapy as the prescription of Anatomical Therapeutic Chemical codes in subgroup B03A (iron preparations), and defined primary care use as the number of consultations in the year preceding inclusion. For the general practitioners, we retrieved sex, age, workload (in consultations per working week), and urbanity of the practice location (according to the Swiss Federal Statistical Office^21^).

### Statistical Analysis

We report variable summaries as numbers (percentages) or medians (IQRs) as appropriate. Missing data were handled using a complete-case approach in regression analyses. We conducted all analyses from October 2, 2023, to May 29, 2024, using the statistical software R, version 4.3.2 (R Foundation for Statistical Computing).^22^

We assessed the association of ferritin cutoff choice with the incidence of iron deficiency diagnoses using the following 3 cutoffs recommended by guidelines and expert panels for the adult general population^4,11,23,24,25,26,27^: 15, 30, and 45 ng/mL. For each cutoff and patient, we defined an iron deficiency event as the first measurement of ferritin concentrations below that cutoff during the study period and calculated patient-time as the number of days from (and including) inclusion to (and including) the earliest of either an iron deficiency event, if any, or the end of the study period. For each ferritin cutoff, we excluded patients with iron deficiency as at least 1 ferritin concentration below that cutoff during the year before inclusion from the respective population at risk. We calculated the total incidence of iron deficiency diagnoses as the number of iron deficiency events per 1000 patient-years. We classified iron deficiency events as indicating nonanemic and anemic iron deficiency when accompanied by hemoglobin concentrations within ±4 weeks excluding and indicating anemia, respectively, and calculated their specific incidences. We report all incidence estimates with exact Poisson mean 95% CIs.^28^

Comeasurement of C-reactive protein (CRP) with ferritin testing is often recommended to rule out systemic inflammation, which may elevate ferritin independently of iron storages.^11,23,25,26^ To address this aspect, we conducted a sensitivity analysis in which we repeated the incidence estimations based only on ferritin tests not accompanied by elevated CRP concentrations (within ±1 week, using reported reference ranges).

To assess the incidence and the determinants of ferritin testing, we chose to treat ferritin tests requested after more than 1 year after a previous ferritin test as new instances of ferritin testing, as done in a previous study of the appropriateness of ferritin retesting.^29^ Therefore, we excluded from this analysis all patients who had received ferritin testing in the year preceding inclusion and defined a ferritin testing event as the first ferritin request during the study period for each of the remaining patients. We calculated patient-time as the number of days from (and including) inclusion to (and including) the earliest of either a ferritin testing event, if any, or the end of the study period and obtained the incidence of ferritin testing as the number of ferritin testing events per 1000 patient-years. We fitted a mixed-effects Cox proportional hazards regression model on the ferritin testing event with patient sex-age strata, primary care use, general practitioner sex and age, general practitioner workload, and practice urbanity as covariates at baseline. Anemia, CKD, IBD, rheumatic disease, congestive heart failure, pregnancy, cancer, PPI therapy, and fatigue were included as time-dependent binary covariates,^30^ defined as present starting from the date of their first identification in the database. To account for the same general practitioner treating multiple patients, we used normally distributed general practitioner–level random intercepts. We used the R package coxme^31^ to fit the models and express the associations of the various covariates with ferritin testing as adjusted hazard ratios (AHRs) with corresponding 95% CIs. We quantify general practitioner–level variation as the median hazard ratio (MHR) derived from a null model without general practitioner–level covariates, reported with a Hall percentile 95% CI obtained from 2000 iterations of a cases bootstrap.^32^ The MHR can be interpreted as the median increase in hazard for ferritin testing in a given patient when randomly selecting 2 general practitioners and comparing the one with a higher propensity to the one with a lower propensity to test for ferritin.^33^

We also assessed requests for additional iron studies accompanying ferritin testing (within ±1 week), considering iron studies often mentioned in international and local clinical guidelines for iron deficiency^4,11^: serum iron, transferrin, transferrin saturation, and soluble transferrin receptor. To further examine concomitant testing, we considered all patients of the study cohort who received ferritin testing during the study period and considered the presence of hemoglobin and CRP accompanying the first ferritin test recorded during the study period as 2 distinct binary outcomes. We fitted mixed-effects logistic regression models using the R package lme4^34^ to assess the associations of each outcome with the same determinants and random effects as for ferritin. Associations are expressed as adjusted odds ratios (AORs) with corresponding 95% CIs.

## Results

**Figure 1.**
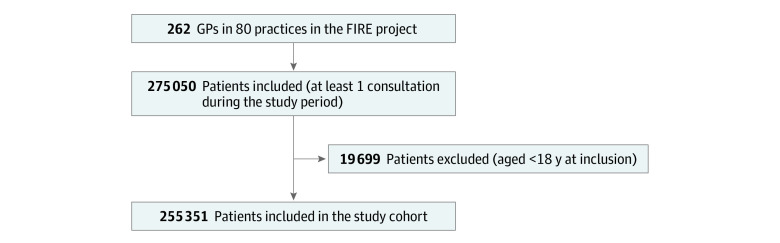
Study Flowchart The study period was January 1, 2021, to November 30, 2023. FIRE indicates Family Medicine Research Using Electronic Medical Records; GP, general practitioner.

The study cohort consisted of 255 351 patients (median [IQR] age, 52 [36-66] years; 52.1% female and 47.9% male). Figure 1 summarizes the cohort selection process, and Table 1 summarizes the patient characteristics. The cohort patients were seen by 262 general practitioners with a median (IQR) age of 48 (40-57) years and a median (IQR) workload of 139 (86-230) consultations per working week in 80 practices. During the study period, 72 817 patients (28.5%) received ferritin testing. Of the first ferritin tests during the study period, 7036 (9.7%) were accompanied by iron studies, most commonly serum iron in 6095 (8.4%), transferrin in 5820 (8.0%), and transferrin saturation in 3856 (5.3%). Accompanying hemoglobin and CRP tests were present in 52 499 (72.1%) and 36 136 (49.6%) cases, respectively.

**Table 1.** Characteristics of the Study Cohort^a^

Characteristic	Did not receive ferritin testing during the study period (n = 182 534)	Received ferritin testing during the study period (n = 72 817)
Patient sex		
Female	83 455 (45.7)	49 543 (68.0)
Male	98 965 (54.2)	23 270 (32.0)
Unknown or other	114 (0.1)	4 (<0.01)
Patient age at inclusion, y		
<25	15 795 (8.7)	5886 (8.1)
25-34	28 116 (15.4)	9441 (13.0)
35-44	29 353 (16.1)	11 000 (15.1)
45-54	29 133 (16.0)	11 216 (15.4)
55-64	25 431 (13.9)	11 335 (15.6)
65-74	25 125 (13.8)	12 364 (17.0)
≥75	29 351 (16.1)	11 573 (15.9)
Unknown	230 (0.1)	2 (<0.01)
History of clinical factors at inclusion		
Anemia	11 767 (6.4)	12 176 (16.7)
Iron therapy	7576 (4.2)	12 269 (16.8)
CKD	6244 (3.4)	5946 (8.2)
IBD	861 (0.5)	826 (1.1)
Rheumatic diseases	1548 (0.8)	1199 (1.6)
CHF	602 (0.3)	436 (0.6)
Pregnancy	712 (0.4)	804 (1.1)
Cancer	2642 (1.4)	2007 (2.8)
PPI therapy	41 954 (23.0)	28 709 (39.4)
Fatigue	2701 (1.5)	3074 (4.2)
No. of consultations in the year prior to inclusion		
0-1	114 529 (62.7)	27 109 (37.2)
2-5	33 802 (18.5)	16 962 (23.3)
>5	34 203 (18.7)	28 746 (39.5)
Received a ferritin test in the year before inclusion	7630 (4.2)	15 884 (21.8)
Characteristics of the first ferritin test during the study period		
Was accompanied by a hemoglobin test	NA	52 499 (72.1)
Was accompanied by a CRP test	NA	36 136 (49.6)
Was accompanied by iron studies		
None	NA	65 781 (90.3)
Serum iron	NA	6095 (8.4)
Transferrin	NA	5820 (8.0)
Transferrin saturation	NA	3856 (5.3)
Soluble transferrin receptor	NA	506 (0.7)
Ferritin concentration, median (IQR), ng/mL	NA	81 (40-155)

Abbreviations: CHF, congestive heart failure; CKD, chronic kidney disease; CRP, C-reactive protein; IBD, inflammatory bowel disease; NA, not applicable; PPI, proton pump inhibitor.

SI conversion factor: To convert ferritin to micrograms per liter, multiply by 1.

^a^ Data are presented as number (percentage) of patients unless otherwise indicated.

Table 2 summarizes the incidences of iron deficiency diagnoses at the different ferritin cutoffs (eTable 3 in Supplement 1). At 15, 30, and 45 ng/mL, the total incidences of iron deficiency diagnoses were 10.9 (95% CI, 10.6-11.2), 29.9 (95% CI, 29.4-30.4), and 48.3 (95% CI, 47.7-48.9) cases per 1000 patient-years, respectively. At 15, 30, and 45 ng/mL, the incidences of nonanemic iron deficiency diagnoses were 4.1 (95% CI, 3.9-4.2), 14.6 (95% CI, 14.3-15.0), and 25.8 (95% CI, 25.3-26.2) cases per 1000 patient-years, respectively, and the incidences of anemic iron deficiency diagnoses were 3.5 (95% CI, 3.3-3.7), 6.0 (95% CI, 5.8-6.2), and 7.5 (95% CI, 7.3-7.7) cases per 1000 patient-years, respectively.

**Table 2.** Incidences of Iron Deficiency Diagnoses (Nonanemic, Anemic, and Total) at Different Serum Ferritin (Ferritin) Cutoffs

Diagnosis	Incidences in cases per 1000 patient-years (95% CI)		
	15 ng/mL	30 ng/mL	45 ng/mL
**Any ferritin test**			
Nonanemic iron deficiency	4.1 (3.9-4.2)	14.6 (14.3-15.0)	25.8 (25.3-26.2)
Anemic iron deficiency	3.5 (3.3-3.7)	6.0 (5.8-6.2)	7.5 (7.3-7.7)
Total iron deficiency	10.9 (10.6-11.2)	29.9 (29.4-30.4)	48.3 (47.7-48.9)
**Only ferritin tests not accompanied by elevated CRP concentrations**			
Nonanemic iron deficiency	4.2 (4.0-4.4)	14.6 (14.3-15.0)	25.6 (25.2-26.0)
Anemic iron deficiency	3.1 (3.0-3.3)	5.3 (5.1-5.5)	6.6 (6.4-6.8)
Total iron deficiency	9.8 (9.6-10.1)	26.7 (26.2-27.1)	43.0 (42.5-43.6)

Abbreviation: CRP, C-reactive protein.

**Figure 2.**
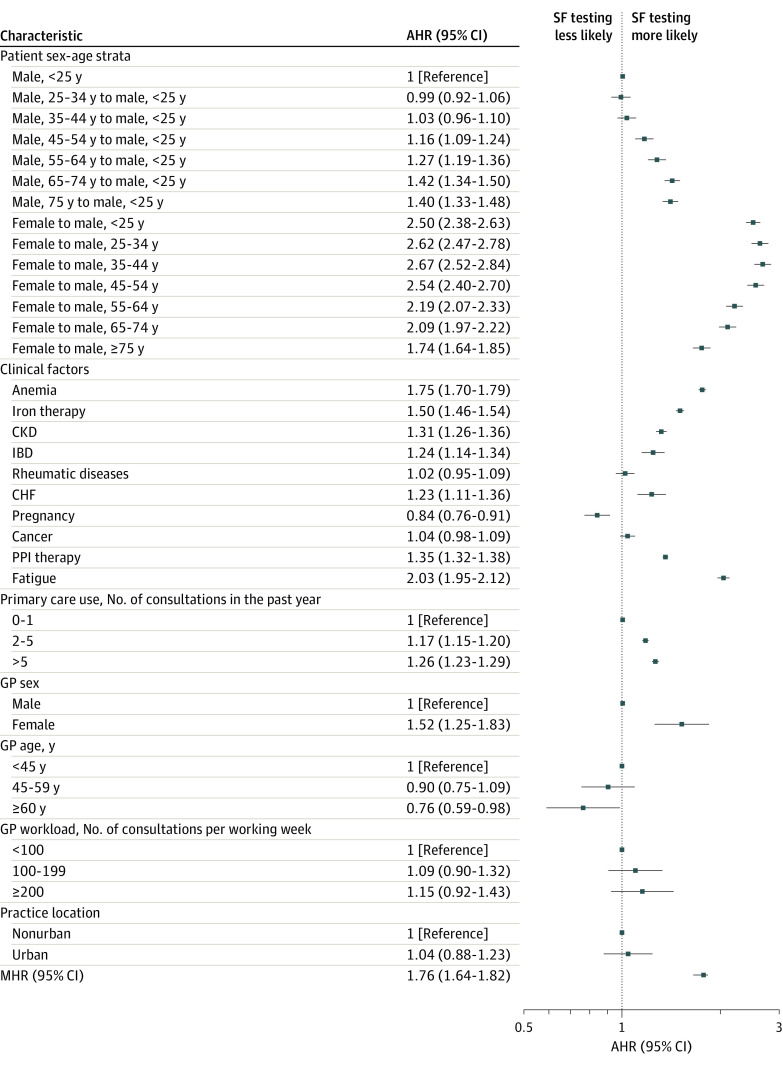
Determinants of Ferritin Testing Results of mixed-effects Cox proportional hazards regression, expressed as adjusted hazard ratios (AHRs) for covariates and a median hazard ratio (MHR, derived from a corresponding null model without general practitioner [GP]–level covariates) to summarize the random-effects distribution. Associations with patient sex-age strata are summarized as follows: for male patients, as AHRs with respect to male patients younger than 25 years; and for female patients, as AHRs with respect to male patients of the respective same age category. A total of 255 119 were studied (to avoid artifacts due to high statistical imbalance, 232 patients were excluded due to unknown or other sex). A total of 56 927 ferritin testing events were studied. CHF indicates congestive heart failure, CKD, chronic kidney disease; IBD, inflammatory bowel disease; PPI proton pump inhibitor; SF, serum ferritin.

Among the 231 837 patients (90.8%) in the study cohort who did not receive ferritin testing in the year preceding inclusion, the incidence was 145.6 ferritin testing events per 1000 patient-years (95% CI, 144.4-146.8; median [IQR] follow-up time, 694 [294-956] days). Figure 2 shows the different associations of ferritin testing derived from the Cox proportional hazards regression model (eTables 4-5 in Supplement 1). Ferritin testing showed notable associations with fatigue (AHR, 2.03; 95% CI, 1.95-2.12), anemia (AHR, 1.75; 95% CI, 1.70-1.79), and iron therapy (AHR, 1.50; 95% CI, 1.46-1.54). Ferritin testing was more common in patients with CKD, IBD, PPI therapy, and higher primary care use but was less common with pregnancy. Female patients were more likely to receive ferritin testing than male patients of the same age in all age categories, including postmenopausal. The MHR among general practitioners was 1.76 (95% CI, 1.64-1.82).

Table 3 summarizes the associations with hemoglobin and CRP tests accompanying ferritin testing (eTable 6 in Supplement 1). Both hemoglobin and CRP tests were positively associated with fatigue and were negatively associated with higher primary care use.

**Table 3.** Determinants of Hemoglobin and C-Reactive Protein Testing Accompanying Ferritin Testing^a^

Determinant	AOR (95% CI)		
	Hemoglobin	C-reactive protein
Patient sex-age strata		
Male, <25 y	1.0 [Reference]	1.0 [Reference]
Male, 25-34 y to male, <25 y	1.20 (0.84-1.72)	0.91 (0.75-1.09)
Male, 35-44 y to male, <25 y	1.32 (0.93-1.89)	0.83 (0.69-0.99)
Male, 45-54 y to male, <25 y	1.11 (0.79-1.57)	0.79 (0.66-0.94)
Male, 55-64 y to male, <25 y	1.24 (0.88-1.73)	0.77 (0.65-0.91)
Male, 65-74 y to male, <25 y	1.34 (0.96-1.88)	0.79 (0.67-0.93)
Male, ≥75 y to male, <25 y	1.80 (1.27-2.53)	0.85 (0.71-1.00)
Female to male, <25 y	0.21 (0.04-1.02)	0.33 (0.13-0.82)
Female to male, 25-34 y	0.91 (0.70-1.18)	1.06 (0.93-1.21)
Female to male, 35-44 y	0.83 (0.64-1.06)	1.12 (0.99-1.27)
Female to male, 45-54 y	1.00 (0.79-1.26)	1.12 (1.00-1.26)
Female to male, 55-64 y	0.89 (0.71-1.11)	1.05 (0.94-1.17)
Female to male, 65-74 y	0.81 (0.65-1.01)	1.06 (0.95-1.18)
Female to male, ≥75 y	1.59 (1.15-2.20)	0.90 (0.76-1.06)
Clinical factors		
Anemia	1.61 (1.32-1.96)	0.95 (0.86-1.05)
Iron therapy	0.72 (0.63-0.82)	0.97 (0.91-1.03)
CKD	1.05 (0.88-1.25)	0.80 (0.74-0.87)
IBD	1.61 (1.10-2.36)	1.13 (0.95-1.35)
Rheumatic diseases	0.79 (0.59-1.07)	1.16 (0.99-1.35)
CHF	0.83 (0.52-1.34)	0.78 (0.63-0.97)
Pregnancy	1.40 (0.92-2.13)	0.91 (0.75-1.10)
Cancer	0.94 (0.74-1.21)	0.97 (0.86-1.09)
PPI therapy	1.09 (0.99-1.20)	0.99 (0.95-1.04)
Fatigue	1.51 (1.22-1.86)	1.15 (1.05-1.27)
Primary care use, No. of consultations in the past year		
0-1	1.0 [Reference]	1.0 [Reference]
2-5	0.83 (0.74-0.93)	0.95 (0.90-1.01)
>5	0.73 (0.65-0.82)	0.86 (0.82-0.91)
GP sex		
Male	1.0 [Reference]	1.0 [Reference]
Female	2.94 (0.93-9.25)	1.58 (0.82-3.04)
GP age, y		
<45	1.0 [Reference]	1.0 [Reference]
45-59	0.64 (0.21-2.02)	0.59 (0.31-1.14)
≥60	1.23 (0.26-5.80)	0.81 (0.33-1.95)
GP workload, No. of consultations per working week		
<100	1.0 [Reference]	1.0 [Reference]
100-199	1.53 (0.43-5.48)	0.73 (0.35-1.51)
≥200	2.23 (0.53-9.47)	0.39 (0.17-0.90)
Practice location		
Nonurban	1.0 [Reference]	1.0 [Reference]
Urban	0.30 (0.11-0.87)	0.51 (0.28-0.93)

Abbreviations: AOR, adjusted odds ratio; CHF, congestive heart failure; CKD, chronic kidney disease; GP, general practitioner; IBD, inflammatory bowel disease; PPI, proton pump inhibitor.

^a^ Associations with patient sex-age strata are summarized as follows: for male patients, as AORs with respect to male patients younger than 25 years; and for female patients, as AORs with respect to male patients of the respective same age category. A total of 72 815 patients were studied (to avoid artifacts due to high statistical imbalance, 2 patients were excluded due to unknown or other sex). A total of 52 499 testing events were studied for hemoglobin and 36 136 for C-reactive protein.

## Discussion

In this study of more than 255 000 patients, we investigated the determinants and variation of ferritin testing and the incidence of iron deficiency diagnoses in Swiss primary care. We observed a substantial association of the choice of ferritin cutoff with the rates of iron deficiency diagnoses, especially nonanemic iron deficiency. In addition, we found a substantial degree of variation in ferritin testing along with interesting associations, such as higher testing rates in postmenopausal women compared with men of the same age and higher testing rates by female and younger general practitioners. We also found gaps in the quality of ferritin testing in terms of a large proportion ordered without accompanying hemoglobin and CRP measurements.

Our most compelling finding regarding the incidence of iron deficiency diagnoses was its strong dependence on the choice of the ferritin cutoff, especially for nonanemic iron deficiency. The use of ferritin cutoffs as treatment thresholds has been widely debated,^35^ and a recent Cochrane review found insufficient evidence to recommend any specific cutoff in a healthy, asymptomatic population.^36^ Even within Switzerland, there are conflicting recommendations. A guideline from a prominent Swiss primary care network emphasizes that iron replacement is not warranted at ferritin concentrations above 15 ng/mL,^11^ whereas a local expert panel has recommended diagnosing iron deficiency at ferritin concentrations below 30 ng/mL.^23^ Our results show that these controversies affect the management of a considerable number of patients.

The observed incidence of anemic iron deficiency diagnoses of approximately 13 cases per 1000 patient-years is comparable to the results of a similar multinational European study.^13^ We are not aware of other studies that have estimated the rates of iron deficiency diagnoses based on routine data in high-resource settings. However, the lack of concomitant hemoglobin and CRP measurements has important implications for the interpretation of such incidences. The proportion of ferritin tests without accompanying CRP or hemoglobin tests was surprisingly high because most guidelines explicitly recommend screening for systemic inflammation and anemia in the workup of iron deficiency.^4^ Hemoglobin and CRP testing was associated with fatigue and with fewer previous primary care visits, suggesting that they were more commonly used in patients in whom a symptom-led search for iron deficiency took place rather than in episodes of care involving routine screening.

Ferritin testing was requested in more than one-fourth of the patients followed up for the 3 years of the study period, which aligns with the findings of a previous Swiss study^8^ and is a rate only slightly higher than previously observed in Australia.^37^ Comparisons of ferritin testing rates with estimates available from other countries, such as the UK^5^ or Canada,^7^ are complicated by the heterogeneity in the reported measures of testing frequencies and of the populations considered. The paucity of comparable international data suggests a need for updated research.

Among the clinical factors considered, fatigue, anemia, and iron therapy showed the strongest associations with ferritin testing. Fatigue was the clinical factor most strongly associated with ferritin testing, but its prevalence of just more than 2% at inclusion was well below previous annual prevalence estimates of approximately 8% in primary care.^38^ This observation is consistent with previous conclusions that the documentation of general symptom-related ICPC-2 codes is likely underrepresented in the FIRE database compared with condition-related ICPC-2 codes.^15^ Nevertheless, our finding suggests that the clinical presentation of the patient plays an important role in the decision to test for ferritin.

Women were invariably more likely to receive ferritin testing than men in the same age group, regardless of anemia or prior iron therapy. Although this sex difference is consistent with a higher risk of iron deficiency due to menstrual blood loss during childbearing age, we have no immediate explanation for this finding in the postmenopausal age group. Symptoms of nonanemic iron deficiency may often have prompted ferritin testing. Many of these symptoms, especially fatigue, are nonspecific but are more prevalent among women as presenting concerns in primary care.^2,39^ Although we adjusted for fatigue, its low frequency of documentation may have resulted in residual confounding that partially explains the persistent association with female sex.

We also observed notable associations of ferritin testing with general practitioner characteristics, with female and younger general practitioners being more prone to ferritin testing. The sex difference can be interpreted in the context of previous findings that female general practitioners provide more preventive care than their male colleagues,^40,41^ and the age disparity may be related to the variation in information-seeking behavior of general practitioners of different ages.^42^ The general practitioner–level variation in ferritin testing expressed by the MHR was striking because it was comparable with several of the AHRs expressing associations with clinical factors. This finding is consistent with previously observed evidence of unwarranted variation in the use of ferritin testing in different primary care settings, which has been interpreted as indicating potential overuse.^6,7,43^

Most ferritin tests were not accompanied by requests for additional iron studies, suggesting awareness among general practitioners of the recommendation to use ferritin as a first-line test for iron deficiency. This result contrasts with findings from other countries, such as Australia^37^ and Spain,^6^ where other iron studies were overrequested compared with ferritin. On the other hand, we observed that serum iron was the most frequently requested additional iron study, contrary to the recommendation of local guidelines to avoid its use.^11^ This finding suggests a knowledge gap regarding the use of iron studies, in line with results from the international literature.^44,45^

In summary, our findings can be understood in the context of clinical uncertainty faced by general practitioners regarding the diagnosis of iron deficiency. This uncertainty may be partly due to the lack of consensus among different recommendations for iron deficiency screening and ferritin cutoffs, ultimately calling for more guidance on the management of iron deficiency in primary care.^46^

### Limitations

This study has some limitations. The FIRE database does not allow access to presenting symptoms as documented in the clinical notes, which is an important limitation together with the lack of information on referral. In addition, we had limited access to information on gastrointestinal risk factors, such as celiac disease. Although information on the patients’ diet, which may have prompted screening for iron deficiency, was also unavailable, a previous study found no relevant differences in the prevalence of iron deficiency among Swiss people who were omnivores, vegetarians, or vegans.^47^

## Conclusions

Our study demonstrates a substantial increase in the rate of iron deficiency diagnoses when ferritin cutoffs of 30 and 45 ng/mL, respectively, are chosen over 15 ng/mL. Our results provide an information base for health system–level evaluations of ferritin testing in primary care. In addition, they highlight the need for harmonization of guidelines for the diagnosis of iron deficiency in primary care.

## Notes

Supplement 1. **eTable 1.** Compiled RECORD (Reporting of Studies Conducted Using Observational Routinely Collected Health Data) checklist **eTable 2.** Operationalized criteria for the identification of clinical characteristics **eTable 3.** Follow-up times, event counts, and incidence of nonanemic, anemic, and total iron deficiency diagnoses at different ferritin cutoffs **eTable 4.** Determinants of ferritin testing (full model) **eTable 5.** Determinants of ferritin testing (null model) **eTable 6.** Determinants of hemoglobin and C-reactive protein testing accompanying ferritin testing

Supplement 2.
Data Sharing Statement
