# Supplementary material for: Ferritin Cutoffs and Diagnosis of Iron Deficiency in Primary Care
Source: JAMA Netw Open. 2024 Aug 5;7(8):e2425692. doi: 10.1001/jamanetworkopen.2024.25692 (PMC11301556; doi:10.1001/jamanetworkopen.2024.25692)
Supplement: Supplement 1. — eTable 1. Compiled RECORD (Reporting of Studies Conducted Using Observational Routinely Collected Health Data) checklist eTable 2. Operationalized criteria for the identification of clinical characteristics eTable 3. Follow-up times, event counts, and incidence of nonanemic, anemic, and total iron deficiency diagnoses at different ferritin cutoffs eTable 4. Determinants of ferritin testing (full model) eTable 5. Determinants of ferritin testing (null model) eTable 6. Determinants of hemoglobin and C-reactive protein testing accompanying ferritin testing [file jamanetwopen-e2425692-s001.pdf]

Supplementary Online Content

Jäger L, Rachamin Y, Seen O, Burgstaller JM, Rosemann T, Markun S. Ferritin cutoffs and diagnosis of iron deficiency in primary care. *JAMA Netw Open*. 2024;7(8):e2425692. doi:10.1001/jamanetworkopen.2024.25692

eTable 1. Compiled RECORD (Reporting Of Studies Conducted Using Observational Routinely Collected Health Data) checklist

eTable 2. Operationalized criteria for the identification of clinical characteristics

eTable 3. Follow-up times, event counts, and incidence of nonanemic, anemic, and total iron deficiency diagnoses at different ferritin cutoffs

eTable 4. Determinants of ferritin testing (full model)

eTable 5. Determinants of ferritin testing (null model)

eTable 6. Determinants of hemoglobin and C-reactive protein testing accompanying ferritin testing

This supplementary material has been provided by the authors to give readers additional information about their work.

eTable 1. Compiled RECORD (Reporting of Studies Conducted Using Observational Routinely Collected Health Data) checklist.

	Item No.	STROBE items	Location in manuscript where items are reported	RECORD items	Location in manuscript where items are reported
**Title and abstract**					
	1	(a) Indicate the study's design with a commonly used term in the title or the abstract (b) Provide in the abstract an informative and balanced summary of what was done and what was found	Abstract	RECORD 1.1: The type of data used should be specified in the title or abstract. When possible, the name of the databases used should be included.<br>RECORD 1.2: If applicable, the geographic region and timeframe within which the study took place should be reported in the title or abstract.<br>RECORD 1.3: If linkage between databases was conducted for the study, this should be clearly stated in the title or abstract.	1.1: Type of data and name of database (FIRE) are included in the Abstract.<br>1.2: Location (Switzerland) and study period are mentioned in the Abstract.<br>1.3: No linkage was conducted.
**Introduction**					
Background rationale	2	Explain the scientific background and rationale for the investigation being reported	Introduction		
Objectives	3	State specific objectives, including any prespecified hypotheses	Introduction (the study was exploratory in nature and did not include any prespecified hypotheses)		
**Methods**					
Study Design	4	Present key elements of study design early in the paper	Methods		
Setting	5	Describe the setting, locations, and relevant dates, including periods of recruitment, exposure, follow-up, and data collection	Methods		
Participants	6	(a) *Cohort study* - Give the eligibility criteria, and the sources and methods of selection of participants. Describe methods of follow-up<br>*Case-control study* - Give the eligibility criteria, and the sources and methods of case ascertainment and control selection. Give the rationale for the choice of cases and controls	(a) This study is a cohort study. Eligibility criteria, selection, and follow-up are described in the Methods.<br>(b) No matching was performed in this study.	RECORD 6.1: The methods of study population selection (such as codes or algorithms used-identify subjects) should be listed in detail. If this is not possible, an explanation should be provided.<br>RECORD 6.2: Any validation studies of the codes or algorithms used-select the population should be referenced. If validation was conducted for this study	6.1: The rule-based selection criteria are defined in the Methods. The rule-based identification criteria for relevant clinical factors are described in eTable2.<br>6.2: Validation studies for the rule-based identification methods are not available.

© 2024 Jäger L et al. *JAMA Network Open.*

	Item No.	STROBE items	Location in manuscript where items are reported	RECORD items	Location in manuscript where items are reported
		Cross-sectional study - Give the eligibility criteria, and the sources and methods of selection of participants<br>(b) *Cohort study* - For matched studies, give matching criteria and number of exposed and unexposed<br>*Case-control study* - For matched studies, give matching criteria and the number of controls per case		and not published elsewhere, detailed methods and results should be provided.<br>RECORD 6.3: If the study involved linkage of databases, consider use of a flow diagram or other graphical display-demonstrate the data linkage process, including the number of individuals with linked data at each stage.	6.3: No linkage was conducted.
Variables	7	Clearly define all outcomes, exposures, predictors, potential confounders, and effect modifiers. Give diagnostic criteria, if applicable.	Methods	RECORD 7.1: A complete list of codes and algorithms used-classify exposures, outcomes, confounders, and effect modifiers should be provided. If these cannot be reported, an explanation should be provided.	The rule-based criteria for explanatory variables (exposures, confounders, and effect modifiers) are described in eTable 2. The criteria for study outcomes are described in the Methods section of the main text.
Data sources / measurement	8	For each variable of interest, give sources of data and details of methods of assessment (measurement).<br>Describe comparability of assessment methods if there is more than one group	Not applicable (all data was retrieved in the same way from one database).		
Bias	9	Describe any efforts-address potential sources of bias	Various potential sources of bias are addressed in the Methods and Discussion.		
Study size	10	Explain how the study size was arrived at	Results, Figure 1 (study flowchart)		
Quantitative variables	11	Explain how quantitative variables were handled in the analyses. If applicable, describe which groupings were chosen, and why	Methods (patient age)		
Statistical methods	12	(a) Describe all statistical methods, including those used-control for confounding<br>(b) Describe any methods used-examine subgroups and interactions<br>(c) Explain how missing data were addressed<br>(d) *Cohort study* - If applicable, explain how loss-follow-up was addressed	(a)-(c): Methods<br>(d) This study is a cohort study. The classical definition of loss-follow-up was not applicable-the study (since it considered events in routine care).<br>(e) Methods		

	Item No.	STROBE items	Location in manuscript where items are reported	RECORD items	Location in manuscript where items are reported
		Case-control study - If applicable, explain how matching of cases and controls was addressed<br>*Cross-sectional study* - If applicable, describe analytical methods taking account of sampling strategy<br>(e) Describe any sensitivity analyses			
Data access and cleaning methods		..		RECORD 12.1: Authors should describe the extent-which the investigators had access-the database population used-create the study population.<br>RECORD 12.2: Authors should provide information on the data cleaning methods used in the study.	12.1: Methods (no access: anonymized database)<br>12.2: Information can be found in the seminal FIRE publication cited in the Methods section.
Linkage		..		RECORD 12.3: State whether the study included person-level, institutional-level, or other data linkage across two or more databases. The methods of linkage and methods of linkage quality evaluation should be provided.	No linkage was conducted.
**Results**					
Participants	13	(a) Report the numbers of individuals at each stage of the study (e.g., numbers potentially eligible, examined for eligibility, confirmed eligible, included in the study, completing follow-up, and analysed)<br>(b) Give reasons for non-participation at each stage.<br>(c) Consider use of a flow diagram	(a), (b): Results, Figure 1<br>(c) Figure 1	RECORD 13.1: Describe in detail the selection of the persons included in the study (i.e., study population selection) including filtering based on data quality, data availability and linkage. The selection of included persons can be described in the text and/or by means of the study flow diagram.	Methods (data availability issues were not relevant, since the database includes all mentioned components from all participating practices).
Descriptive data	14	(a) Give characteristics of study participants (e.g., demographic, clinical, social) and information on exposures and potential confounders<br>(b) Indicate the number of participants with missing data for each variable of interest<br>(c) *Cohort study* - summarise follow-up time (e.g., average and total amount)	(a) Results<br>(b) Table 1<br>(c) Results, eTable 3		

	Item No.	STROBE items	Location in manuscript where items are reported	RECORD items	Location in manuscript where items are reported
Outcome data	15	*Cohort study* - Report numbers of outcome events or summary measures over time<br>*Case-control study* - Report numbers in each exposure<br>category, or summary measures of exposure<br>*Cross-sectional study* - Report numbers of outcome events or summary measures	This study is a cohort study. Summaries of outcome events and summary measures are reported in the Results and in Table 1.		
Main results	16	(a) Give unadjusted estimates and, if applicable, confounder-adjusted estimates and their precision (e.g., 95% confidence interval). Make clear which confounders were adjusted for and why they were included<br>(b) Report category boundaries when continuous variables were categorized<br>(c) If relevant, consider translating estimates of relative risk into absolute risk for a meaningful time period	(a) Results (association estimates), Methods (inclusion of covariates)<br>(b) Methods, Table 1<br>(c) Not applicable		
Other analyses	17	Report other analyses done—e.g., analyses of subgroups and interactions, and sensitivity analyses	Methods, Results (interaction of sex and age; sensitivity analysis involving C-reactive protein)		
**Discussion**					
Key results	18	Summarise key results with reference-study objectives	Discussion		
Limitations	19	Discuss limitations of the study, taking into account sources of potential bias or imprecision. Discuss both direction and magnitude of any potential bias	Discussion	RECORD 19.1: Discuss the implications of using data that were not created or collected-answer the specific research question(s). Include discussion of misclassification bias, unmeasured confounding, missing data, and changing eligibility over time, as they pertain-the study being reported.	Discussion
Interpretation	20	Give a cautious overall interpretation of results considering objectives, limitations, multiplicity of analyses,	Discussion		

© 2024 Jäger L et al. *JAMA Network Open.*

	Item No.	STROBE items	Location in manuscript where items are reported	RECORD items	Location in manuscript where items are reported
		results from similar studies, and other relevant evidence			
Generalisability	21	Discuss the generalisability (external validity) of the study results	Discussion		
**Other Information**					
Funding	22	Give the source of funding and the role of the funders for the present study and, if applicable, for the original study on which the present article is based	Funding disclosure		
Accessibility of protocol, raw data, and programming code		..		RECORD 22.1: Authors should provide information on how-access any supplemental information such as the study protocol, raw data, or programming code.	Data availability statement

Abbreviations: FIRE, Family medicine Research using Electronic medical records project; STROBE, STrengthening the Reporting of OBservational studies in Epidemiology; RECORD, REporting of studies Conducted using Observational Routinely-collected health Data.

© 2024 Jäger L et al. *JAMA Network Open.*

eTable 2. Operationalized criteria for the identification of clinical characteristics.

Clinical characteristic	Criteria
Anemia	Hemoglobin concentrations below 12 g/L for women and 13 g/L for men.[1]
Iron therapy	Prescription of any ATC code in subgroup B03A (Iron preparations).
Chronic kidney disease	At least two estimated glomerular filtration rate values (calculated from serum creatinine concentrations according-the CKD-EPI equation[2]) < 60 mL/min/1.73m[2] at least 3 months apart.[3]
Inflammatory bowel disease	Any of the following:<br>– Prescription of any GTIN in the PCG “Morbus Crohn / Colitis ulcerosa”.<br>– ICPC-2 code D94 (Chronic enteritis/ulcerative colitis).
Rheumatic diseases	Any of the following:<br>– Prescription of any GTIN in the PCG “Rheuma”.<br>– ICPC-2 code L88 (Rheumatoid/seropositive arthritis).
Congestive heart failure	Any of the following:<br>– Prescription of ATC code C09DX04 (Valsartan and sacubitril).<br>– ICPC-2 code D94 (Chronic enteritis/ulcerative colitis).
Pregnancy	Any ICPC-2 code in group W (Pregnancy, Childbearing, Family Planning) except W01 (Question of pregnancy), W02 (Fear of pregnancy), W10 (Contraception postcoital), W11 (Contraception oral), W12 (Contraception intrauterine), W13 (Sterilization), W14 (Contraception other), W15 (Infertility/subfertility).
Cancer	Any of the following:<br>– Prescription of any GTIN in the PCGs “Krebs” or “Krebs complex”.<br>– ICPC-2 codes A79 (malignancy NOS), B72 (Hodgkin’s disease/lymphoma), B73 (Leukaemia), B74 (Malignant neoplasm blood other), D74 (Malignant neoplasm stomach), D75 (Malignant neoplasm colon/rectum), D76 (Malignant neoplasm pancreas), D77 (Malignant neoplasm digestive other/NOS), L71 (Malignant neoplasm musculoskeletal), N74 (Malignant neoplasm nervous system), R84 (Malignant neoplasm bronchus/lung), R85 (Malignant neoplasm respiratory, other), T71 (Malignant neoplasm thyroid), U75 (Malignant neoplasm of kidney), U76 (Malignant neoplasm of bladder), U77 (Malignant neoplasm urinary other), W72 (Malignant neoplasm related-pregnancy), X75 (Malignant neoplasm cervix), X76 (Malignant neoplasm breast female), X77 (Malignant neoplasm genital other (f)), Y77 (Malignant neoplasm prostate), Y78 (Malignant neoplasm male genital other).
Proton-pump inhibitor therapy	Prescription of any ATC code in subgroup A02BC (Proton pump inhibitors).

Abbreviations: ATC, Anatomic Therapeutic Chemical classification system[4]; GTIN, Global Trade Item Number; ICPC-2, International Classification of Primary Care, 2nd edition[5]; NOS, Not Otherwise Specified; PCG, Pharmaceutical Cost Group.[6]

© 2024 Jäger L et al. *JAMA Network Open.*

eTable 3. Follow-up times, event counts, and incidence of nonanemic, anemic, and total iron deficiency diagnoses at different ferritin cutoffs.

		**Any ferritin test**		
		**15 ng/mL**	**30 ng/mL**	**45 ng/mL**
	No. of persons at risk	253777	250975	248265
	Follow-up in days, median (IQR)	885 (581-1015)	864 (534-1010)	843 (497-1005)
**Nonanemic iron deficiency**	No. of events	2156	7496	12753
	Incidence per 1000 patient-years (95% CI)	4.1 (3.9-4.2)	14.6 (14.3-15.0)	25.8 (25.3-26.2)
**Anemic iron deficiency**	No. of events	1866	3060	3708
	Incidence per 1000 patient-years (95% CI)	3.5 (3.3-3.7)	6.0 (5.8-6.2)	7.5 (7.3-7.7)
**Total iron deficiency**	No. of events	5793	15324	23889
	Incidence per 1000 patient-years (95% CI)	10.9 (10.6-11.2)	29.9 (29.4-30.4)	48.3 (47.7-48.9)
		**Only ferritin tests not accompanied by elevated CRP concentrations**		
		**15 ng/mL**	**30 ng/mL**	**45 ng/mL**
	No. of persons at risk	253704	251193	248776
	Follow-up in days, median (IQR)	886 (584-1015)	869 (541-1010)	849 (507-1007)
**Nonanemic iron deficiency**	No. of events	2234	7541	12777
	Incidence per 1000 patient-years (95% CI)	4.2 (4.0-4.4)	14.6 (14.3-15.0)	25.6 (25.2-26.0)
**Anemic iron deficiency**	No. of events	1654	2710	3303
	Incidence per 1000 patient-years (95% CI)	3.1 (3.0-3.3)	5.3 (5.1-5.5)	6.6 (6.4-6.8)
**Total iron deficiency**	No. of events	5246	13756	21480
	Incidence per 1000 patient-years (95% CI)	9.8 (9.6-10.1)	26.7 (26.2-27.1)	43.0 (42.5-43.6)

Total iron deficiency diagnoses are defined as ferritin concentrations below the respective cutoff, regardless of accompanying hemoglobin measurements. Nonanemic and anemic iron deficiency refer-ferritin concentrations below the respective cutoff accompanied by hemoglobin concentrations indicating and excluding anemia, respectively. For each cutoff, we excluded patients with iron deficiency as defined by at least one ferritin concentration below that respective cutoff during the year prior-the patient's inclusion. Note that the size of the population at risk varies accordingly for each cutoff. Abbreviations: CI, confidence interval, CRP, C-reactive protein; IQR, interquartile range.

© 2024 Jäger L et al. *JAMA Network Open.*

eTable 4. Determinants of ferritin testing (full model).

Covariate	Beta (SE)	AHR (95% CI)
Patient sex-age strata		
Male, 25-34 years-male, < 25 years	-0.01 (0.03)	0.99 (0.92-1.06)
Male, 35-44 years-male, < 25 years	0.03 (0.03)	1.03 (0.96-1.10)
Male, 45-54 years-male, < 25 years	0.15 (0.03)	1.16 (1.09-1.24)
Male, 55-64 years-male, < 25 years	0.24 (0.03)	1.27 (1.19-1.36)
Male, 65-74 years-male, < 25 years	0.35 (0.03)	1.42 (1.34-1.50)
Male, ≥ 75 years-male, < 25 years	0.34 (0.03)	1.40 (1.33-1.48)
Female-male, < 25 years	0.92 (0.03)	2.50 (2.38-2.63)
Female-male, 25-34 years	0.96 (0.03)	2.62 (2.47-2.78)
Female-male, 35-44 years	0.98 (0.03)	2.67 (2.52-2.84)
Female-male, 45-54 years	0.93 (0.03)	2.54 (2.40-2.70)
Female-male, 55-64 years	0.79 (0.03)	2.19 (2.07-2.33)
Female-male, 65-74 years	0.74 (0.03)	2.09 (1.97-2.22)
Female-male, ≥ 75 years	0.55 (0.03)	1.74 (1.64-1.85)
Female, 25-34 years-female, < 25 years*	-0.10 (0.02)	0.90 (0.86-0.94)
Female, 35-44 years-female, < 25 years*	-0.09 (0.02)	0.92 (0.88-0.96)
Female, 45-54 years-female, < 25 years*	-0.13 (0.02)	0.87 (0.84-0.91)
Female, 55-64 years-female, < 25 years*	-0.28 (0.02)	0.75 (0.72-0.79)
Female, 65-74 years-female, < 25 years*	-0.33 (0.02)	0.72 (0.69-0.75)
Female, ≥ 75 years-female, < 25 years*	-0.51 (0.02)	0.60 (0.57-0.63)
Male-female, < 25 years*	-1.07 (0.03)	0.34 (0.32-0.37)
Male-female, 25-34 years*	-0.98 (0.03)	0.38 (0.36-0.40)
Male-female, 35-44 years*	-0.95 (0.02)	0.39 (0.37-0.40)
Male-female, 45-54 years*	-0.78 (0.02)	0.46 (0.44-0.48)
Male-female, 55-64 years*	-0.54 (0.02)	0.58 (0.56-0.61)
Male-female, 65-74 years*	-0.40 (0.02)	0.67 (0.64-0.70)
Male-female, ≥ 75 years*	-0.19 (0.02)	0.83 (0.79-0.86)
Anemia	0.56 (0.01)	1.75 (1.70-1.79)
Iron therapy	0.40 (0.01)	1.50 (1.46-1.54)
CKD	0.27 (0.02)	1.31 (1.26-1.36)
IBD	0.21 (0.04)	1.24 (1.14-1.34)
Rheumatic diseases	0.02 (0.03)	1.02 (0.95-1.09)
CHF	0.20 (0.05)	1.23 (1.11-1.36)
Pregnancy	-0.18 (0.05)	0.84 (0.76-0.91)
Cancer	0.03 (0.03)	1.04 (0.98-1.09)
PPI therapy	0.30 (0.01)	1.35 (1.32-1.38)
Fatigue	0.71 (0.02)	2.03 (1.95-2.12)
Primary care utilization (consultations in the past year; reference: 0-1)		
2-5	0.16 (0.01)	1.17 (1.15-1.20)
> 5	0.23 (0.01)	1.26 (1.23-1.29)
Female GP	0.42 (0.10)	1.52 (1.25-1.83)
GP age (reference: < 45 years)		
45-59 years	-0.10 (0.10)	0.90 (0.75-1.09)
≥ 60 years	-0.28 (0.13)	0.76 (0.59-0.98)

© 2024 Jäger L et al. *JAMA Network Open.*

Covariate	Beta (SE)	AHR (95% CI)
GP workload (consultations/working week; reference: < 100)		
100-199	0.09 (0.10)	1.09 (0.90-1.32)
≥ 200	0.14 (0.11)	1.15 (0.92-1.43)
Urban practice location	0.04 (0.09)	1.04 (0.88-1.23)

Results of a mixed-effects Cox proportional hazards regression model with both patient- and GP-level covariates. The table contains results for associations of serum ferritin testing with (fixed-effect) model covariates as coefficient estimates (beta) with standard errors (SEs) and as adjusted hazard ratios (AHRs) with 95% confidence intervals (CIs). Number of patients: 255119 (to avoid artifacts due-high statistical imbalance, 232 patients were excluded due-unknown or other sex). Number of ferritin testing events: 56927. *These comparisons are an alternative way of expressing the associations with sex-age strata. Abbreviations: CHF, congestive heart failure; CKD, chronic kidney disease; GP, general practitioner; IBD, inflammatory bowel disease; PPI, proton-pump inhibitor.

© 2024 Jäger L et al. *JAMA Network Open.*

eTable 5. Determinants of ferritin testing (null model).

Covariate	Beta (SE)	AHR (95% CI)
Patient sex-age strata		
Male, 25-34 years-male, < 25 years	-0.01 (0.03)	0.99 (0.93-1.06)
Male, 35-44 years-male, < 25 years	0.03 (0.03)	1.03 (0.97-1.10)
Male, 45-54 years-male, < 25 years	0.15 (0.03)	1.16 (1.09-1.24)
Male, 55-64 years-male, < 25 years	0.24 (0.03)	1.27 (1.20-1.36)
Male, 65-74 years-male, < 25 years	0.35 (0.03)	1.41 (1.34-1.49)
Male, ≥ 75 years-male, < 25 years	0.33 (0.03)	1.39 (1.32-1.47)
Female-male, < 25 years	0.92 (0.03)	2.50 (2.38-2.63)
Female-male, 25-34 years	0.97 (0.03)	2.64 (2.49-2.80)
Female-male, 35-44 years	0.98 (0.03)	2.68 (2.52-2.84)
Female-male, 45-54 years	0.93 (0.03)	2.55 (2.40-2.70)
Female-male, 55-64 years	0.78 (0.03)	2.19 (2.06-2.32)
Female-male, 65-74 years	0.74 (0.03)	2.09 (1.97-2.22)
Female-male, ≥ 75 years	0.55 (0.03)	1.74 (1.64-1.85)
Female, 25-34 years-female, < 25 years*	-0.10 (0.02)	0.91 (0.87-0.95)
Female, 35-44 years-female, < 25 years*	-0.09 (0.02)	0.92 (0.88-0.96)
Female, 45-54 years-female, < 25 years*	-0.14 (0.02)	0.87 (0.84-0.91)
Female, 55-64 years-female, < 25 years*	-0.29 (0.02)	0.75 (0.72-0.78)
Female, 65-74 years-female, < 25 years*	-0.33 (0.02)	0.72 (0.68-0.75)
Female, ≥ 75 years-female, < 25 years*	-0.52 (0.02)	0.60 (0.57-0.63)
Male-female, < 25 years*	-1.07 (0.03)	0.34 (0.32-0.37)
Male-female, 25-34 years*	-0.98 (0.03)	0.37 (0.36-0.39)
Male-female, 35-44 years*	-0.95 (0.02)	0.39 (0.37-0.40)
Male-female, 45-54 years*	-0.78 (0.02)	0.46 (0.44-0.48)
Male-female, 55-64 years*	-0.54 (0.02)	0.58 (0.56-0.61)
Male-female, 65-74 years*	-0.40 (0.02)	0.67 (0.64-0.70)
Male-female, ≥ 75 years*	-0.19 (0.02)	0.83 (0.79-0.86)
Anemia	0.56 (0.01)	1.75 (1.71-1.79)
Iron therapy	0.40 (0.01)	1.50 (1.46-1.54)
CKD	0.27 (0.02)	1.31 (1.26-1.36)
IBD	0.22 (0.04)	1.24 (1.14-1.34)
Rheumatic diseases	0.02 (0.03)	1.02 (0.95-1.09)
CHF	0.21 (0.05)	1.23 (1.11-1.36)
Pregnancy	-0.18 (0.05)	0.83 (0.76-0.91)
Cancer	0.04 (0.03)	1.04 (0.98-1.09)
PPI therapy	0.30 (0.01)	1.35 (1.33-1.38)
Fatigue	0.71 (0.02)	2.03 (1.95-2.12)
Primary care utilization (consultations in the past year; reference: 0-1)		
2-5	0.16 (0.01)	1.17 (1.15-1.20)
> 5	0.23 (0.01)	1.26 (1.23-1.29)

Results of a mixed-effects Cox proportional hazards regression model only with patient-level covariates. The table contains results for associations of serum ferritin testing with (fixed-effect) model covariates as coefficient estimates (beta) with standard errors (SEs) and as adjusted hazard ratios (AHRs) with 95% confidence intervals (CIs). Number of patients: 255119 (to avoid artifacts due-high statistical imbalance, 232 patients were excluded due-unknown or other sex). Number of ferritin testing events: 56927. *These comparisons are an alternative way of expressing the associations with sex-age strata. Abbreviations: CHF, congestive heart failure; CKD, chronic kidney disease; IBD, inflammatory bowel disease; PPI, proton-pump inhibitor.

© 2024 Jäger L et al. *JAMA Network Open.*

eTable 6. Determinants of hemoglobin and C-reactive protein testing accompanying ferritin testing.

	Hemoglobin		C-reactive protein	
**Covariate**	**Beta (SE)**	**AOR (95% CI)**	**Beta (SE)**	**AOR (95% CI)**
Patient sex-age strata (Reference: Male, < 25 years)				
Male, 25-34 years-male, < 25 years	0.18 (0.18)	1.20 (0.84-1.72)	-0.10 (0.09)	0.91 (0.75-1.09)
Male, 35-44 years-male, < 25 years	0.28 (0.18)	1.32 (0.93-1.89)	-0.19 (0.09)	0.83 (0.69-0.99)
Male, 45-54 years-male, < 25 years	0.11 (0.18)	1.11 (0.79-1.57)	-0.24 (0.09)	0.79 (0.66-0.94)
Male, 55-64 years-male, < 25 years	0.21 (0.17)	1.24 (0.88-1.73)	-0.26 (0.09)	0.77 (0.65-0.91)
Male, 65-74 years-male, < 25 years	0.29 (0.17)	1.34 (0.96-1.88)	-0.24 (0.09)	0.79 (0.67-0.93)
Male, ≥ 75 years-male, < 25 years	0.59 (0.17)	1.80 (1.27-2.53)	-0.17 (0.09)	0.85 (0.71-1.00)
Female-male, <25 years	-1.58 (0.82)	0.21 (0.04-1.02)	-1.10 (0.46)	0.33 (0.13-0.82)
Female-male, 25-34 years	-0.10 (0.13)	0.91 (0.70-1.18)	0.06 (0.07)	1.06 (0.93-1.21)
Female-male, 35-44 years	-0.19 (0.13)	0.83 (0.64-1.06)	0.11 (0.06)	1.12 (0.99-1.27)
Female-male, 45-54 years	0.00 (0.12)	1.00 (0.79-1.26)	0.11 (0.06)	1.12 (1.00-1.26)
Female-male, 55-64 years	-0.12 (0.11)	0.89 (0.71-1.11)	0.05 (0.06)	1.05 (0.94-1.17)
Female-male, 65-74 years	-0.21 (0.11)	0.81 (0.65-1.01)	0.06 (0.05)	1.06 (0.95-1.18)
Female-male, ≥ 75 years	0.47 (0.17)	1.59 (1.15-2.20)	-0.10 (0.08)	0.90 (0.76-1.06)
Female, 25-34 years-female, < 25 years*	0.05 (0.11)	1.05 (0.85 to 1.29)	0.00 (0.05)	1.00 (0.90 to 1.11)
Female, 35-44 years-female, < 25 years*	0.05 (0.10)	1.06 (0.86 to 1.29)	-0.04 (0.05)	0.96 (0.87 to 1.07)
Female, 45-54 years-female, < 25 years*	0.07 (0.10)	1.07 (0.87 to 1.31)	-0.08 (0.05)	0.92 (0.83 to 1.02)
Female, 55-64 years-female, < 25 years*	0.06 (0.11)	1.06 (0.86 to 1.31)	-0.18 (0.05)	0.84 (0.75 to 0.93)
Female, 65-74 years-female, < 25 years*	0.05 (0.11)	1.05 (0.84 to 1.31)	-0.14 (0.06)	0.87 (0.78 to 0.97)
Female, ≥ 75 years-female, < 25 years*	0.43 (0.12)	1.54 (1.22 to 1.93)	-0.07 (0.06)	0.94 (0.83 to 1.05)
Male-female, < 25 years*	-1.69 (0.80)	0.18 (0.04 to 0.89)	-0.99 (0.45)	0.37 (0.15 to 0.90)
Male-female, 25-34 years*	0.10 (0.13)	1.10 (0.85 to 1.43)	-0.06 (0.07)	0.94 (0.83 to 1.08)
Male-female, 35-44 years*	0.19 (0.13)	1.21 (0.94 to 1.55)	-0.11 (0.06)	0.89 (0.79 to 1.01)
Male-female, 45-54 years*	0.00 (0.12)	1.00 (0.80 to 1.26)	-0.11 (0.06)	0.89 (0.80 to 1.00)
Male-female, 55-64 years*	0.12 (0.11)	1.12 (0.90 to 1.40)	-0.05 (0.06)	0.95 (0.85 to 1.07)
Male-female, 65-74 years*	0.21 (0.11)	1.23 (0.99 to 1.54)	-0.06 (0.05)	0.94 (0.85 to 1.05)
Male-female, ≥ 75 years*	0.55 (0.13)	1.73 (1.34 to 2.24)	-0.13 (0.06)	0.88 (0.78 to 1.00)
Anemia	0.48 (0.10)	1.61 (1.32-1.96)	-0.05 (0.05)	0.95 (0.86-1.05)
Iron therapy	-0.33 (0.07)	0.72 (0.63-0.82)	-0.03 (0.03)	0.97 (0.91-1.03)
CKD	0.05 (0.09)	1.05 (0.88-1.25)	-0.22 (0.04)	0.80 (0.74-0.87)
IBD	0.48 (0.20)	1.61 (1.10-2.36)	0.12 (0.09)	1.13 (0.95-1.35)
Rheumatic diseases	-0.23 (0.15)	0.79 (0.59-1.07)	0.15 (0.08)	1.16 (0.99-1.35)
CHF	-0.18 (0.24)	0.83 (0.52-1.34)	-0.25 (0.11)	0.78 (0.63-0.97)
Pregnancy	0.34 (0.21)	1.40 (0.92-2.13)	-0.09 (0.10)	0.91 (0.75-1.10)
Cancer	-0.06 (0.13)	0.94 (0.74-1.21)	-0.03 (0.06)	0.97 (0.86-1.09)
PPI therapy	0.09 (0.05)	1.09 (0.99-1.20)	-0.01 (0.02)	0.99 (0.95-1.04)
Fatigue	0.41 (0.11)	1.51 (1.22-1.86)	0.14 (0.05)	1.15 (1.05-1.27)
Primary care utilization (consultations in the past year; reference: 0-1)				
2-5	-0.18 (0.06)	0.83 (0.74-0.93)	-0.05 (0.03)	0.95 (0.90-1.01)
> 5	-0.31 (0.06)	0.73 (0.65-0.82)	-0.15 (0.03)	0.86 (0.82-0.91)
Female GP	1.08 (0.58)	2.94 (0.93-9.25)	0.45 (0.34)	1.58 (0.82-3.04)
GP age (reference: < 45 years)				
45-59 years	-0.44 (0.58)	0.64 (0.21-2.02)	-0.53 (0.33)	0.59 (0.31-1.14)
≥ 60 years	0.20 (0.79)	1.23 (0.26-5.80)	-0.22 (0.45)	0.81 (0.33-1.95)

© 2024 Jäger L et al. *JAMA Network Open.*

	Hemoglobin		C-reactive protein	
**Covariate**	**Beta (SE)**	**AOR (95% CI)**	**Beta (SE)**	**AOR (95% CI)**
GP workload (consultations/working week; reference: < 100)				
100-199	0.43 (0.65)	1.53 (0.43-5.48)	-0.32 (0.37)	0.73 (0.35-1.51)
≥ 200	0.80 (0.74)	2.23 (0.53-9.47)	-0.94 (0.42)	0.39 (0.17-0.90)
Urban practice location	-1.19 (0.53)	0.30 (0.11-0.87)	-0.67 (0.30)	0.51 (0.28-0.93)

Results of mixed-effects logistic regression models. The table contains results for associations of hemoglobin and C-reactive protein testing with (fixed-effect) model covariates as coefficient estimates (beta) with standard errors (SEs) and as adjusted odds ratios (AORs) with 95% confidence intervals (CIs). Number of patients: 72815 (to avoid artifacts due-high statistical imbalance, 2 patients were excluded due to unknown or other sex). Number of testing events: 52499 for hemoglobin and 36136 for C-reactive protein. *These comparisons are an alternative way of expressing the associations with sex-age strata. Abbreviations: CHF, congestive heart failure; CKD, chronic kidney disease; GP, general practitioner; IBD, inflammatory bowel disease; PPI, proton-pump inhibitor.

© 2024 Jäger L et al. *JAMA Network Open.*

eReferences

1. World Health Organization. *Haemoglobin concentrations for the diagnosis of anaemia and assessment of severity*. World Health Organization; 2011. https://apps.who.int/iris/handle/10665/85839. Accessed February 9, 2024.

2. Levey AS, Stevens LA, Schmid CH, Zhang YL, Castro Alejandro F 3rd, Feldman HI, et al. *A new equation to estimate glomerular filtration rate*. Ann Intern Med. 2009;150(9):604-612.

3. Eknoyan G, Lameire N, Eckardt K, Kasiske B, Wheeler D, Levin A, et al. *KDIGO 2012 clinical practice guideline for the evaluation and management of chronic kidney disease*. Kidney Int. 2013;3(1):5-14.

4. World Health Organization Collaborating Centre for Drug Statistics Methodology. *ATC classification index with DDDs*. 2023. https://www.whocc.no/. Accessed January 4, 2024.

5. World Organization of National Colleges, Academies and Academic Associations of General Practitioners/Family Physicians (WONCA). *ICPC-2-R: International Classification of Primary Care*. Oxford University Press; 2005.

6. Federal Office of Public Health (Health and Accident Insurance Directorate Premiums/Solvency Oversight Section). *Liste der pharmazeutischen Kostengruppen (PCG-Liste)*. 2023. https://www.bag.admin.ch/bag/en/home/versicherungen/krankenversicherung/krankenversicherung-versicherer-aufsicht/risikoausgleich.html. Accessed February 19, 2024.
