# Supplementary material for: Ferritin Cutoffs and Diagnosis of Iron Deficiency in Primary Care
Source: JAMA Netw Open. 2024 Aug 5;7(8):e2425692. doi: 10.1001/jamanetworkopen.2024.25692 (PMC11301556; doi:10.1001/jamanetworkopen.2024.25692)
Supplement: Supplement 2. — Data Sharing Statement [file jamanetwopen-e2425692-s002.pdf]

Data Sharing Statement

Jäger. Ferritin Cutoffs and Diagnosis of Iron Deficiency in Primary Care. *JAMA Netw Open*. Published August 05, 2024. doi:10.1001/jamanetworkopen.2024.25692

Data
Data available: No

Additional Information
Explanation for why data not available: Individuals or organizations with access to data sets that overlap with data in the FIRE database (such as health insurance claims data) may be able to identify patients in violation of legal restrictions on the identifiability of research subjects in Switzerland. Data access requests can be sent to the corresponding author or to the FIRE research group (fire@usz.ch) at the University of Zurich.
